# *Leptothrix cholodnii* Response to Nutrient Limitation

**DOI:** 10.3389/fmicb.2021.691563

**Published:** 2021-06-24

**Authors:** Tatsuki Kunoh, Tatsuya Yamamoto, Shinya Sugimoto, Erika Ono, Nobuhiko Nomura, Andrew S. Utada

**Affiliations:** ^1^Faculty of Life and Environmental Sciences, University of Tsukuba, Tsukuba, Japan; ^2^Department of Bacteriology, Jikei Center for Biofilm Research and Technology, The Jikei University School of Medicine, Minato-ku, Japan; ^3^School of Life and Environmental Sciences, University of Tsukuba, Tsukuba, Japan; ^4^Microbiology Research Center for Sustainability, University of Tsukuba, Tsukuba, Japan

**Keywords:** filamentous bacterium, *Leptothrix*, Microfluidics, nutrient limitation, sheath formation

## Abstract

Microorganisms are widely utilized for the treatment of wastewater in activated sludge systems. However, the uncontrolled growth of filamentous bacteria leads to bulking and adversely affects wastewater treatment efficiency. To clarify the nutrient requirements for filament formation, we track the growth of a filamentous bacterium, *Leptothrix cholodnii* SP-6 in different nutrient-limited conditions using a high aspect-ratio microfluidic chamber to follow cell-chain elongation and sheath formation. We find that limitations in Na^+^, K^+^, and Fe^2+^ yield no observable changes in the elongation of cell chains and sheath formation, whereas limitations of C, N, P, or vitamins lead to more pronounced changes in filament morphology; here we observe the appearance of partially empty filaments with wide intercellular gaps. We observe more dramatic differences when SP-6 cells are transferred to media lacking Mg^2+^ and Ca^2+^. Loss of Mg^2+^ results in cell autolysis, while removal of Ca^2+^ results in the catastrophic disintegration of the filaments. By simultaneously limiting both carbon and Ca^2+^ sources, we are able to stimulate planktonic cell generation. These findings paint a detailed picture of the ecophysiology of *Leptothrix*, which may lead to improved control over the unchecked growth of deleterious filamentous bacteria in water purification systems.

## Introduction

Sustainable management of water resources is an issue of fundamental importance for supporting the health of growing populations and continued development of economic activity. Microorganisms are widely employed in water purification and wastewater treatment plants, where their innate biological function and metabolism are harnessed to remove contaminants from surface water and ground water as well as urban wastewater such as industrial drainage. Several Fe-/Mn-oxidizing bacteria, such as the *Leptothrix* and *Gallionella* species, are commonly used to make water potable due to their ability to remove dissolved metals from surface water and ground water through biomineralization (Mouchet, 1992; Demir, 2016). Their biosorption activity for arsenic, phosphorous, and transition metals is currently being studied for use in wastewater treatment industries (Katsoyiannis and Zouboulis, 2006; Kunoh et al., 2017b; Buliauskaite et al., 2020).

In contrast to the usefulness of bacteria for industrial water filtration and wastewater treatment, filamentous bacteria such as various species of *Sphaerotilus*, *Thiothrix*, and *Leptothrix*, can grow exponentially, thereby forming microbial mats that clog water distribution systems and lead to bulking and/or foaming in activated sludge tanks of wastewater treatment plants (van Veen et al., 1978; Kunoh et al., 2016a; Henriet et al., 2017). As they grow, these filamentous bacteria generate woven mesh-like networks that clog pipes and reduce the efficiency of separating activated sludge flocs from the water column (Eggerichs et al., 2020). This reduced efficiency ultimately results in lower quality clarified water. To temporarily suppress bulking and foaming, treatment facilities rely on chemical treatment with Cl_2_, ozone, H_2_O_2_, Al salts and other “catch-all” methods (D’Antoni et al., 2017); however, they do not address the causes of the excessive growth of filamentous bacteria. More research is needed to understand how these bacteria grow to identify possible methods of mitigation. To this end, developing a fundamental understanding of the mechanism of filament formation employed by such bacteria could identify new control techniques that disrupt or inhibit this mechanism, halting growth when needed to improve the capacity and efficiency of wastewater treatment while limiting the use of toxic chemicals.

*Leptothrix* is a genus of filamentous bacteria that ubiquitously inhabit Fe-rich ground water seeps and streams, where they generate macroscopic mat-like biofilms composed of randomly interwoven filaments. They are also inhabitants in water purification plants and activated sludge in wastewater treatment plants (van der Waarde et al., 2002; Sawayama et al., 2011). In industrial settings, these mats can lead to clogging. *Leptothrix* mat development proceeds through several stages beginning with the irreversible attachment of planktonic cells to solid surfaces mediated by hair-like appendages called nanofibrils (van Veen et al., 1978; Kunoh et al., 2016a, 2020). Under favorable conditions, cell division generates a chain of daughter cells that are gradually wrapped in a self-secreted sheath of extracellular polysaccharides (EPS) that imposes linear alignment. Environmental fluid flow then causes aggregation of these filaments, while filament elongation interweaves different filaments, creating a porous network (Kunoh et al., 2016a). *Leptothrix* oxidation of dissolved Fe and Mn produces metal oxide nanoparticles that adsorb to the sheath matrix resulting in a rigid, rust-colored, organic/inorganic hybrid tube structure (Corstjens et al., 1992; Emerson and Ghiorse, 1992). These rigid “microtubes” provide structural support to the porous networks (Furutani et al., 2011). Subsequent aggregation generates larger flocs, which hierarchically aggregate, forming mats. These porous environments serve an important ecological role by providing habitable infrastructure that enables the migration of other species and nutrient sharing (Chan et al., 2016).

Microbial mat formation is a macroscopic process that is driven by filament aggregation, while filament formation is, itself, a process which is driven by aggregation of components that comprise the sheath. The sheath is a randomly interwoven matrix of EPS nanofibrils, which are glycoconjugate nanofibers that contain thiol, carboxyl, and amino functional groups (Takeda et al., 2005). The thiol groups are critical for crosslinking nanofibrils, which strengthens the sheath (Emerson and Ghiorse, 1993a; Spring, 2006), whereas the amino groups facilitate the adsorption of oxide nanoparticles in the sheath matrix (Kunoh et al., 2017a). During the course of filament elongation, the nanofibrils in older sections of the filament become interwoven into a sheath surrounding the cells, whereas in newest sections near elongating filament tips, the secreted nanofibrils remain loose and unbraided. Due to the cohesiveness of the sheath, elongating filaments rarely break into shorter filaments, even when they collide with obstacles or produce an intercellular gap in aligned cell chain (Kunoh et al., 2020). However, the disorganization of the sheath structure of the distal pole of the filament leaves cells only partially constrained by nanofibrils; this allows some cells to escape. It appears that sheath maturation coupled to filament elongation is an important feature that balances the retainment of cells in the filament versus the release of planktonic cells that will nucleate new filaments.

The sheaths are constructed using materials acquired from the local environment, so the availability of key nutrients impacts sheath development and maturation. For example, sheath maturation does not proceed in the absence of Fe and Mn (Furutani et al., 2011). This characteristic provides a lever that could be used to manage the growth of *Leptothrix* through the regulation of the locally dissolved minerals; however, the relationship between the abundance of nutrients and filament development remains unclear. The origins of the changes in nutrient availability could be driven by changes in the local environment or the result of targeted efforts to reduce the nutrient. In the case of *Leptothrix*, a detailed picture of the relationship between nutrient availability and filament formation could have important ramifications for the control and management of microbial mats in industrial settings.

In this paper, we investigate the effect of abrupt depletion of key nutrients on filamentous growth of *L. cholodnii* SP-6 which is formerly classified as *L. discophora* (Emerson and Ghiorse, 1993b; Emerson et al., 2010). Since several *Leptothrix* species including *L. discophora* present in bulking activated sludge in industrial wastewater treatment plants (van der Waarde et al., 2002), we use SP-6 as a model filament-forming organism. Although concentrations of divalent cations Mg^2+^ and/or Ca^2+^ are known to interfere with growth of SP-6 (Eggerichs et al., 2020), requirement of nutrients for sheath development remains elusive. We thus use a high-aspect ratio microfluidic chamber to enable visualization of sheath development during filament elongation under various nutrient-depleted conditions and atmospheric scanning electron microscopy (ASEM) to image development of porous network to identify changes based on nutrient conditions. We find that there are at least four distinct morphological changes to filamentous growth in *L. cholodnii* cells that result from limitations in carbon, nitrogen, phosphorous, vitamins, Mg^2+^, and Ca^2+^. Calcium limitation, in particular, so severely affects sheath development that during growth, filaments frequently splinter into shorter fragments. We utilize this fact to show that filament formation is fully arrested when we limit both carbon and Ca^2+^. Our findings clarify an important control mechanism of the filamentous growth of *Leptothrix*, which may contribute to the development of technologies that can suppress clogging of water distribution systems and bulking and foaming in wastewater treatment plants due to filamentous bacteria.

## Materials and Methods

### Bacteria Strains, Media, and Culturing Methods

In this study, we used *L. cholodnii* strains SP-6 (ATCC 51168) (Emerson and Ghiorse, 1993b) purchased from ATCC and OUMS1 (NITE BP-860) (Sawayama et al., 2011) generously provided by Prof. Jun Takada (Okayama Univ., Japan). Herein, we referred to as SP-6 and OUMS1, respectively. Cells were transferred from freezer stocks to a MSVP agar plate (Supplementary Tables 1, 2; Emerson and Ghiorse, 1993b) and incubated at room temperature (RT). We preferred to employ MSVP, a synthetic complete medium, by which we were able to deplete a target nutritional element accurately and thus we could evaluate the effects of such a depletion on the filamentous growth. MSVP is known to support exponential growth of SP-6 cell filaments encased in a sheath (Emerson and Ghiorse, 1993b) and contains mineral salts, vitamins, and sodium pyruvate. After culturing for 7 days, we transferred 1–3 single colonies to 25 mL of the original MSVP in 50 mL conical tube (Iwaki, Tokyo, Japan) and incubated them in a reciprocating shaker at RT and 65 rpm for an additional 2 days. Referring to the growth curve under the same culture condition, the growth stage of 2-day liquid culture corresponds to the mid-log phase (Kunoh et al., 2016a). Following liquid culture, cells were used immediately. MSVP severely depleted in a carbon source (MSVP-C), a nitrogen source (MSVP-N), vitamins (MSVP-V), magnesium (MSVP-Mg), iron (MSVP-Fe), calcium (MSVP-Ca), and phosphorus (MSVP-P) were prepared by omitting sodium pyruvate, (NH)_2_SO_4_, vitamin stock, MgSO_4_ • 7H_2_O, FeSO_4_ • 7H_2_O, CaCl_2_ • 2H_2_O, KH_2_PO_4_, and Na_2_HPO_4_, respectively, during preparation. For MSVP-K, KH_2_PO_4_ was replaced with NaH_2_PO_4_. In MSVP-Na, we replaced sodium pyruvate, Na_2_HPO_4_, and NaOH with potassium pyruvate, K_2_HPO_4_, and KOH, respectively. For MSVP-Ca(NH_4_Cl), CaCl_2_.2H_2_O was replaced with NH_4_Cl. Since incubation of *Leptothrix* cells in ultrapure water results in immediate autolysis (Kunoh et al., 2015), we used 4-(2-hydroxyethyl)-1-piperazineethanesulfonic acid (HEPES) to stabilize pH and maintain osmotic pressure of the media and do not account for its presence when we generate nutrient depleted media in this study. When preparing MSVP-C, MSVP-N, MSVP-Ca, and MSVP-Ca(NH_4_Cl), we ignored the trace amounts (<0.0005 mM) of C, N, and Ca found in the vitamin stock because the concentrations of sodium pyruvate, ammonium sulfate, and calcium chloride dihydrate, which were used as C, N, and Ca sources in unmodified MSVP, respectively, are all greater than 0.4 mM (Supplementary Tables 1, 2). We made a separate calcium-deficient medium by adding the Ca^2+^ chelator ethylene glycol tetraacetic acid (EGTA) to the original MSVP at a final concentration of 3 mM (hereafter MSVP + EGTA). Note that EGTA was not added for Ca^2+^ depletion in MSVP-Ca, MSVP-Ca(NH_4_Cl) and also MSVP-C-Ca. To obtain ASEM snapshots without changing the medium, we also incubated SP-6 cells in a modified MSVP medium containing one-tenth the amount of sodium pyruvate (referred to as MSVP-lowC) that was supplied to maintain minimal filamentous growth. For reference, 2×YPG medium (Bocioaga et al., 2014) treated or untreated with EGTA were similarly used to examine whether the filament breaks induced by the EGTA treatment were medium-dependent or not.

### *In situ* Culturing in a Microfluidic Device

To facilitate imaging of individual cell filaments, we cultured SP-6 cells using a microfluidic device that contains high aspect ratio chambers with the dimensions of 100 μm × 100 μm × ∼1.3 μm. The low ceiling height causes cells to grow “in-plane” while preventing cells or filaments from growing over one another. We have referred to these as 2D chambers (Kunoh et al., 2020). The culture was infused into the microfluidic device using a positive displacement syringe pump (Harvard Apparatus, Holliston, MA, United States) at a rate of 100 μL/h. We inoculated a sufficient amount of 2-day culture into the main channel to achieve a seeding density of approximately 1∼10 cells per 2D chamber. Images were taken on an Axio Observer.Z1 (Carl Zeiss, Oberkochen, Germany) or an SR-10 (Olympus, Tokyo, Japan) at intervals varying from 5–300 s. These microscopes are equipped with a heating unit capable of maintaining a stable temperature of 25°C. We determined the total length of the cell filaments in an individual image picked up from the time-lapse image sequence (every 30 min from the starting time of imaging) using the measuring tool in PhotoShop (Adobe, San Jose, CA, United States). When tips of elongating cell filaments collide to the chamber wall, they change the elongating direction by bending or reversal (Kunoh et al., 2020). Accordingly, to exclude the effects of collision on the filament elongation, we stopped measuring filament lengths when elongating tips were impeded (MSVP, MSVP-K, MSVP-Na at *t* = 10 h; MSVP-P at *t* = 12 h; and MSVP-V at *t* = 16 h) (see Figure 2B and Supplementary Figure 3B).

**FIGURE 1:**
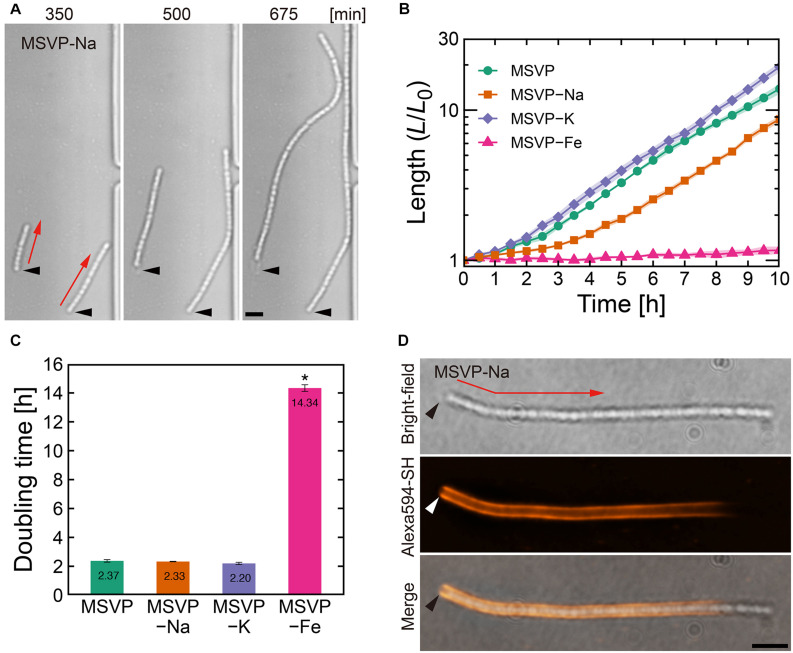
Filament elongation in the normal mode. **(A)** Time-lapse image sequence showing filamentous growth in MSVP-Na. The start time (*t* = 0) is defined as when the first cell makes initial surface attachment. **(B)** Average length of three independent cell filaments measured as a function of time in MSVP, MSVP-Na, MSVP-K, and MSVP-Fe. The filament length (*L*) is normalized by the initial length (*L*_0_) at *t* = 0. Filled regions represent the standard deviation (SD) from three experiments. **(C)** Doubling time of filament length during exponential growth in MSVP, MSVP-Na, MSVP-K, and MSVP-Fe, respectively, measured from three cells. The error bars represent the SD. The significant difference compared to growth in MSVP is calculated using Dunnet’s test (*n* = 3, *P < 0.0000001). **(D)** Fluorescent labeling of the mature sheath surrounding a cell filament grown in MSVP-Na. Black and white arrowheads indicate the same spatial position in each image while red arrows indicate the direction of elongation. Scale bars = 5 μm.

**FIGURE 2:**
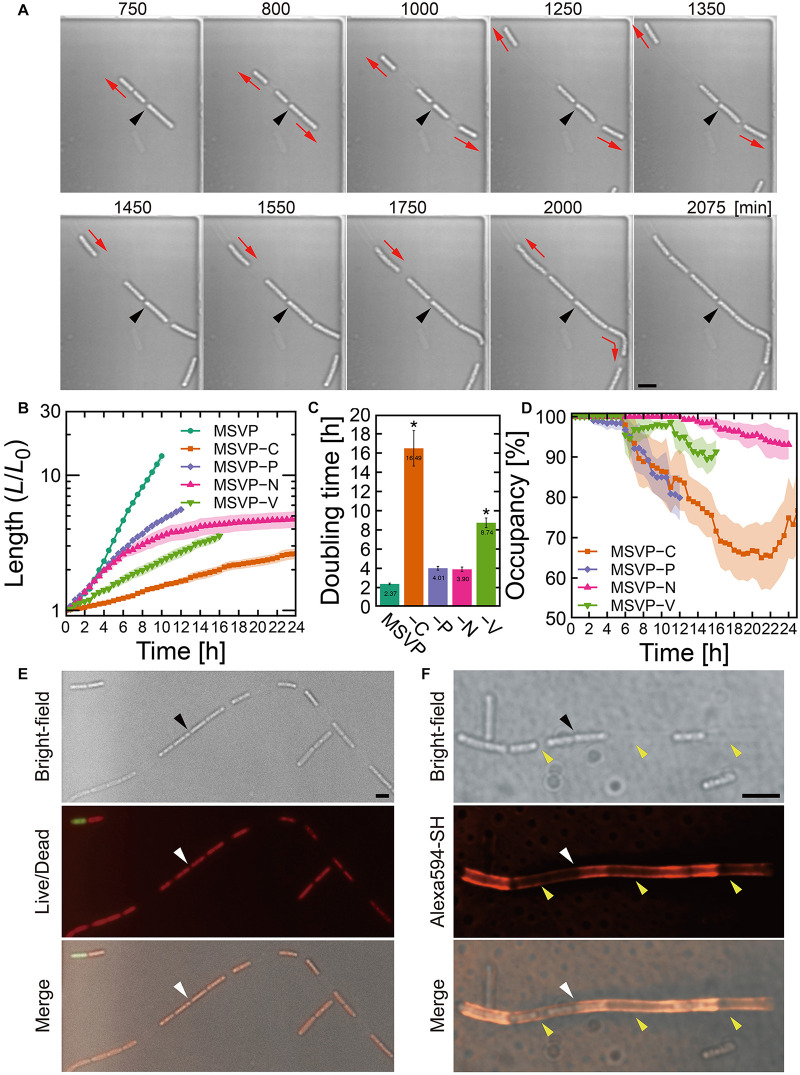
Cell filament elongation in the patchy mode. **(A)** Time-lapse image sequence showing filamentous growth in MSVP-C. Initial surface attachment of the cells is defined as *t* = 0. Black arrowheads indicate the same spatial position in each frame and red arrows indicate the direction of the cell filament elongation. **(B)** Summed length of all cells in the filament measured from three independent cell filaments normalized by their initial length and grown in MSVP, MSVP-C, MSVP-P, MSVP-N, and MSVP-V. Filled region represents one SD from three experiments. **(C)** Doubling time during exponential growth in MSVP, MSVP-C, MSVP-P, MSVP-N, and MSVP-V. The error bars represent the SD (*n* = 3). The significant difference compared to growth in MSVP is caluculated using Dunnet’s test (*n=3*, *P < 0.002). **(D)** Average occupancy (dotted line) and SD (filled region) of three independent filaments filled with cells as a function of time when grown on MSVP-C, MSVP-P, MSVP-N, and MSVP-V. **(E)** Live/dead cell labeling. Red fluorescence labels live cells while green fluorescence labels dead cells. **(F)** Fluorescent labeling of the sheath in MSVP-C. Black and white arrowheads in **(E,F)** indicate the same spatial positions in each frame, while yellow arrowheads in **(F)** indicate the positions of wide intercellular gaps. Scale bars = 5 μm.

### Fluorescent Staining of Nanofibril Thiol Groups and Live/Dead Cells

To visualize the distribution of nanofibrils and maturation of the sheath (Takeda et al., 2012), we stained nanofibrils with Alexa Fluor 594 C5-maleimide (Alexa 594-SH, Thermo Fisher Scientific, Waltham, MA, United States) at a concentration of 30 μM. We first inoculated the cells into the 2D chambers and tracked cell-filament elongation by infusing MSVP. We then added 500 μL of media containing Alexa 594-SH and simultaneously stopped the flow. This mixture was incubated in the microfluidic channels at 25°C for 15 min. We then restarted the flow of MSVP to wash out the unbound reagent and commenced fluorescence imaging. We performed Live/Dead staining with a mixture of SYTOX Green (1:4,000 dilution) and SYTO 59 (1:1,000 dilution) nucleic acid stains (Thermo Fisher Scientific) for 15 min. SYTO 59 permeates all cells, whereas SYTOX Green does not cross the membranes of live cells.

### Imaging of Cell Filament Aggregation in Culture Dishes

To image aggregated cell filaments, we pipetted 600 μL of SP-6 culture into a polymer coverslip bottom dish (μ-Dish 35 mm Quad, Ibidi GmbH, Gräfelfing, Germany) and imaged with an LSM780 confocal microscope (Carl Zeiss) at 15 min intervals. We imaged thin film-like aggregates at the air-liquid interface by incubating 2 mL of culture in a 24-well dish (Iwaki, Tokyo, Japan) using an Axio Zoom.V16 microscope (Carl Zeiss) equipped with a heater unit (Tokai Hit, Fujinomiya, Japan) at 15 min intervals. To image the aggregates at higher magnifications, we scooped the bacterial film and placed it on coverslips.

### Atmospheric Scanning Electron Microscopy (ASEM)

We used ASEM (JASM-6200, JEOL, Tokyo, Japan) to directly visualize development of porous network (Kunoh et al., 2020). We inoculated the cells in the original MSVP into an ASEM membrane dish (35 mm in diameter, JEOL, Tokyo, Japan) by transferring 3 mL of 2-day culture. After a 2 h incubation, the planktonic cells were removed by one medium change and further incubated for additional 16–18 h. The medium was then replaced with MSVP + EGTA, which we define as the starting point of the experiment (*t* = 0 h). After ≈4 h of incubation, the cells were washed in sterile phosphate-buffered saline (PBS), and the remaining surface-attached cells were fixed with 1% (v/v) glutaraldehyde. After quenching in 50 mM ammonium chloride, the nanofibrils secreted from cell surfaces were visualized by staining with positively charged gold nanoparticles (Nanoprobes, Yaphank, NY, United States). We then treated the gold nanoparticles with gold enhancement (GoldEnhance, Nanoprobes), followed by staining in 2% (v/v) phosphotungstic acid (TAAB Laboratories Equipment, Aldermaston, Berkshire, England). Finally, specimens were soaked in 1% (v/v) ascorbic acid and observed using ASEM at an acceleration voltage of 30 kV (Sugimoto et al., 2016).

### Statistics

All statistical analyses were performed using R^1^ and its package “multcomp” (Hothorn et al., 2008).

## Results and Discussion

### SP-6 Cell Filament Formation in Nutrient-Limited Media

The dispersal of cells from typical environmental biofilms depends strongly on local environmental factors, including nutrient levels, microbial growth rate, gene expression profiles, availability of multivalent cations, and quorum-sensing signals (Applegate and Bryers, 1991; Peyton and Characklis, 1993: Allison et al., 1998; Jackson et al., 2002; Kierek and Watnick, 2003). In the case of SP-6, previous report show that cell escape occurs from the elongating pole(s) of filaments due to the local immaturity of the sheath (Kunoh et al., 2020). However, it remains unclear why certain cells at the leading pole are able to escape while others are retained to form a new section of the filament. We hypothesize that altering the local environmental conditions, such as by abruptly lowering the availability of dissolved nutrients or minerals could affect *Leptothrix* behavior, leading to a change in the frequency of cell escape.

To measure the effect of abrupt nutrient limitation on cell behavior, we culture SP-6 cells in 2D chambers that are fed with MSVP culture media that are severely depleted in specific components. As a baseline, we confirm that *L. cholodnii* cultured in full strength MSVP display behavior that is consistent with previous reports (see Supplementary Movie 1 and Supplementary Figure 1; Kunoh et al., 2020). Based on our observations, we classify cell filament development into the following four modes: (i) “normal,” where the filament elongates without the appearance of intercellular gaps; (ii) “patchy,” where large intercellular gaps appear within the filaments; (iii) “non-viable,” where cell autolysis occurs before cell division can proceed; and (iv) “fragmented,” where the cell filaments frequently split into smaller fragments during elongation.

### Filament Elongation and Sheath Maturation in the Normal Mode

In comparison with filaments cultured in MSVP that exhibit the normal mode, we discern no obvious changes in the morphology of elongating cell filaments cultured in MSVP-Na, MSVP-K, and MSVP-Fe as shown in Figure 1A (see Supplementary Figures 2A, 3A and Supplementary Movies 2–4). We find that filaments elongate exponentially in MSVP-Na and MSVP-K, similar to full strength MSVP medium, as shown in Figure 1B. In contrast, while the apparent structure of the filament remains the same, the rate of filament elongation in MSVP-Fe decreases dramatically due to a seven-fold increase in doubling time of filament length cultured in this medium, as shown in Figure 1C (see Supplementary Figure 3B). In the environment, *L. ochracea* and *L. cholodnii* OUMS1 cells grow plentifully in well-pumped groundwater that contains relatively low concentrations of dissolved sodium and potassium (0.45 mM Na^+^ and 0.046 mM K^+^) (Suzuki et al., 2012; Hashimoto et al., 2014). Thus, *Leptothrix* cells are seemingly able to stock sufficient intracellular quantities for normal growth for the duration of the experiments. Alternatively, since it has been reported that in other bacteria K^+^ may be largely replaced with NH^4+^, having minimal effect on growth (Buurman et al., 1989), the same may be true for *Leptothrix*.

Contrasting sharply with these results, we find that Fe^2+^ limitation clearly and immediately slows cell growth, which is consistent with the suggestions from a previous study (Kunoh et al., 2016a). Although it is known that *L. cholodnii* require no more than trace amounts of Fe^2+^ for growth (van Veen et al., 1978; Kunoh et al., 2016a), our results clarify the fact that the rate of filament elongation is strongly affected by the lack of Fe^2+^ (Figures 1B,C and Supplementary Figure 3B). The presence of structural changes in the sheaths would imply differences between nutrient-rich and nutrient-poor conditions; however, the homogeneity of the sheaths of filaments grown in MSVP-Fe versus full strength MSVP are similar as is seen in the fluorescent staining patterns (Supplementary Figure 3C). In this mode, fluorescently labeled cell filaments acquire the strongest fluorescence in the vicinity of the initial attachment point of the founder cell, while the elongating pole(s) remain unlabeled (Kunoh et al., 2020). The monotonic decrease in fluorescence from the initial attachment point toward the elongating pole is a measure of sheath maturation in the direction of filament growth, as shown in Figure 1D (see Supplementary Figures 1C, 2B; Kunoh et al., 2020). Based on these results, we conclude that limiting Na^+^, K^+^, or Fe^2+^ appears to impact neither sheath development nor filament formation other than a suppression of the cellular division rate in the case of MSVP-Fe.

### Intercellular Gap Formation in Sheathed Cell Filaments in the “Patchy” Mode

In contrast to the normal mode, surface-attached cells grown in MSVP-C, MSVP-N, MSVP-P, and MSVP-V form filaments that develop large intercellular gaps, as shown in Figure 2A (see Supplementary Figure 4 and Supplementary Movies 5–8). In these media, we frequently observe that the cell at the distal tip of the filament creates a track of EPS as it moves forward, which allows the trailing cells to follow and appears to define the full extent of the cell filament (Figure 2A, *t* = 1550 min). To characterize these incompletely filled filaments, we define their true length as the summed length of all cells that originate from the founder cell. We find that the normalized length (*L*/*L*_0_) of these patchy filaments is considerably shorter than filaments grown in MSVP (Figure 2B). One reason for this is the significant decrease in filament length is due to the large increase in cellular doubling time in these media (Figure 2C). Interestingly, the dynamics of the change in doubling time of filament length suggests that certain nutrients can either be stored intracellularly or are not needed for a few hours after the medium is switched. For example, depletion of N and P has little effect on growth for ≈4 h, whereas the depletion of vitamins and C immediately increases the doubling time. In the case of MSVP-C, the doubling time increases ∼seven-fold relative to MSVP. Since C, N, and P are major components of the cells of all living organisms, while vitamins are frequently required as growth factors in many bacteria (Peterson and Peterson, 1945), it is not surprising that cell growth slows considerably and the phenotype of filamentation changes in these nutrient-limited media (Fagerbakke et al., 1996; Elser et al., 2000; Vrede et al., 2002). To describe the dynamics of the intercellular gap formation within the filaments, we plot the percent occupancy in the filament as a function of time. We find that in each of these media, after an initial period of relatively normal growth, gaps appear, which lowers the percent occupancy. Depletion of C and P produces the largest drops in percent occupancy, as shown in Figure 2D (see Supplementary Figure 5).

Despite the decrease in overall filament length relative to growth in MSVP, we observe that filament elongation continues even after intercellular gaps appear in these nutrient depleted media. This suggests that the individual cells are still active, which we confirm with live/dead staining in MSVP-C (Figure 2E). Since sheath maturation is driven by the cells in the filament, we image the effect of the intercellular gaps on sheath development by fluorescently labeling it. Like the irregular cell distribution in the filament, we find an irregular fluorescence distribution in the sheath, as shown in Figure 2F (see Supplementary Figure 6). This irregular distribution of fluorescence intensity contrasts with the monotonic decrease in fluorescence intensity as observed in the normal mode (Supplementary Figure 1C) and is particularly evident in filaments grown in C-limited medium. However, despite failure for holding aligned cell chain, the sheath remains sufficiently robust to prevent cell escape from the middle of the filament despite having a heterogeneous thickness due to the irregular sheath development.

In addition to the heterogeneity of sheath development, carbon limitation most strongly affects filament growth relative to the other depleted nutrients (nitrogen, phosphorus, and vitamins) that cause the patchy mode. Here, we specifically focus on limiting carbon to investigate its effect on development of porous network, using ASEM. In these experiments, instead of a complete removal of carbon, which would prevent the cells from proliferating, we culture SP-6 in “low carbon” medium (MSVP-lowC) to generate sufficient numbers of filamentous cells for imaging. Our ASEM images show that filaments grown in MSVP-lowC develop numerous high-curvature bends (Supplementary Figure 7); this suggests that they are softer than filaments grown in full strength MSVP. The apparent decrease in rigidity is likely due, in part, to the fact that the filament is only partially filled with cells when cultured in MSVP-lowC, whose presence can increase the filament stiffness (Vesenka et al., 2018). These results indicate that despite limitations in important nutrients that lead to incompletely filled and mechanically weaker filaments, relative to control conditions, sheath formation still occurs and prevents most cells from escaping the filament.

### Autolysis and Ablation of Sheath Formation in Mg^2+^- and Ca^2+^-Depleted Media

**FIGURE 3:**
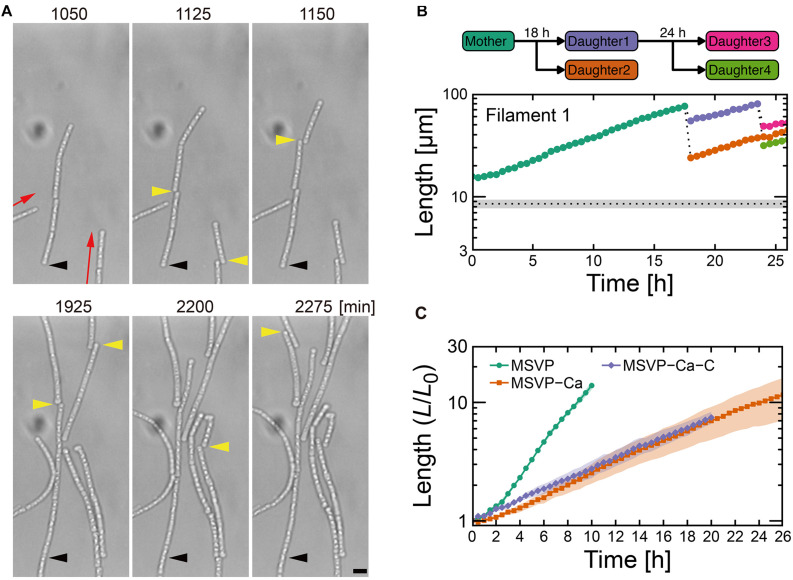
Cell filament elongation in the fragmentation mode in MSVP-Ca. **(A)** Time-lapse sequence showing filamentous growth in MSVP-Ca after surface attachment (*t* = 0). Black and yellow arrowheads indicate the same spatial positions and breaking points in each frame, respectively. Red arrows indicate the direction of elongation. Scale bars = 5 μm. **(B)** The length increase measured as a function of time of a mother filament and the splintered filaments after fragmentation. Black dots line indicates the average length of cells immediately divided in MSVP. (also see Supplementary Figures 9A,B). **(C)** Average length of all cells originated from three independent cell filaments measured as a function of time in MSVP, MSVP-Ca, and MSVP-Ca-C (purple). The filament length (*L*) is normalized by the initial length (*L*_0_) at *t* = 0. Filled regions represent the standard deviation (SD) from three experiments.

In contrast to the normal and patchy modes, we observe more dramatic differences when SP-6 cells are transferred to media lacking Mg^2+^ or Ca^2+^, respectively. In MSVP-Mg, cells that attach in the chambers exhibit a “non-viable” mode where they lyse within a few hours of attachment without dividing (Supplementary Figure 8 and Supplementary Movie 9). This result is consistent with the fact that divalent cations, particularly Mg^2+^, play an important role in maintaining the stability of the outer membrane in most bacteria (Groisman et al., 2013). In contrast, when cells are transferred to MSVP-Ca, after a period of normal but slower filament elongation compared to culture in MSVP, the filaments begin splintering into fragments of random lengths, as shown in Figure 3A (see Supplementary Movie 10). We find that most filament fracturing occurs for times greater than ∼15 h after initial attachment. The resultant multicellular fragments continue to elongate at nearly the same rate as the mother filament and are also subject to splintering as shown in Figure 3B (see Supplementary Figures 9A,B). Importantly, we note that the smaller multicellular filaments do not appear to become single cells through fragmentation.

In addition to the frequent splintering, we observe that when elongating filaments collide with the chamber wall, the pre-impact shape is deflected during filament bending (Supplementary Figure 9C). This is unlike what is observed for filaments cultured in full strength MSVP: as the filament bends due to the collision, the pre-impact shape of the filaments remains unchanged because the filament is supported by attachments to the floor and ceiling of the device (Kunoh et al., 2020). This subtle difference in pre- and post-bending shape indicates weaker adhesion between the sheath and the chamber in MSVP-Ca, which allows it to be pushed as the filament tip bends.

The frequent splintering and the weaker surface adhesion of the filament suggests that sheath formation is hindered in Ca-depleted conditions. Consistent with this picture, fluorescent labeling of cells cultured in MSVP-Ca for *t* > ≈10 h reveals that the short cell fragments lack any visible sheath, while the oldest sections of the mother filament are covered with a mature sheath (Supplementary Figure 9D), which is likely an artifact of how we culture the cells. We believe that prior to transferring SP-6 cells to MSVP-Ca, the individual cells have already begun generating a sheath in full strength MSVP. Then, post transfer, the cells experience a calcium deficit that precludes their ability to produce new sheath and thus upon division, the filament begins to fragment. These results are consistent with a previous study on the filamentous bacterium *Sphaerotilus*, which showed that individual cells were unable to form filaments in Ca-limited medium (Dias et al., 1968). Moreover, they point to the importance of dissolved Ca^2+^ for sheath development and suggest a possible method for control.

### Fragmentation Induced by Addition of EGTA

We prepare MSVP-Ca by omitting CaCl_2_ from components of the full strength MSVP (Supplementary Table 1). Although cell filaments cause fracturing in this medium as shown in Figure 3, we still suspect that this phenomenon can be ascribed to lack of chloride. We thus examine this suspicion by following two ways without changing the chloride concentration: (i) using another Ca^2+^-depleted medium MSVP-Ca(NH_4_Cl) in which CaCl_2_ is substituted with NH_4_Cl as described in Eggerichs et al. (2020) and (ii) using MSVP + EGTA medium in which a calcium chelator, EGTA, is supplemented. We culture SP-6 cells in MSVP-Ca(NH_4_Cl) in polymer coverslip bottom dishes and track filament growth. Supplementary Figure 10 indicates that cell filaments frequently break in MSVP-Ca(NH_4_Cl) but not in control MSVP. Next, we track filament growth in MSVP for 12 h before replacing the medium with MSVP + EGTA. We find that within 5 min of infusing MSVP + EGTA into the microfluidic device, a few filaments splinter into shorter filaments of almost equal length, whereas in other filaments, intercellular gaps appear followed by filament splintering. Time-lapse image sequences of the filaments pre- and post-infusion of MSVP + EGTA and the filament length plotted as function of time are shown in Supplementary Figures 11A–D (see Supplementary Movies 11A,B). After this fragmentation, the majority of splintered filaments continue to elongate exponentially regardless of their breaking. From these results, we conclude that the loss of Ca^2+^ from the environment induces filament fragmentation but has little effect on the rate of filament elongation.

To visualize the effect on sheath development, we fluorescently label the sheaths ≈4 h after the infusion of MSVP + EGTA to allow the filaments time to grow in the calcium depleted medium. The images show that the region of the cell filament that made initial surface-attachment are encased in a sheath, whereas new growth lacks any visible sheath (Supplementary Figure 11E). This indicates that the sheath structure biosynthesized prior to addition of EGTA remains intact. We again turn to ASEM to directly image development of porous network cultured with and without exogenously added EGTA. In the ASEM images, we find that short filaments are rare when we image immediately after addition of EGTA, whereas after 4 h of incubation with EGTA we find a greater abundance of shorter filaments (Supplementary Figure 11F). Since Ca^2+^ deficiency appears to slow or stop sheath formation, cells near the elongating pole(s) of the filaments may be more likely to escape from the filament, which may be the cause for the increase in number of cells not associated with a filament.

To exclude the possibility that the characteristics of SP-6 in Ca^2+^-depleted MSVP are medium-dependent or not. We culture SP-6 cells in 2×YPG medium in polymer coverslip bottom dishes. In 2×YPG supplemented with EGTA, the cell filaments frequently break into small fragments, while in the EGTA-untreated medium, the cell filaments continue to grow without splintering (Supplementary Figure 12). These results strongly suggest that the characteristics induced by Ca^2+^-depletion are not medium-dependent.

From these results, we conclude that Ca^2+^ is required for filamentation in SP-6 cells. Calcium ions are essential for biofilm formation through the direct interaction with exopolysaccharides, providing mechanical strength for the biofilms in other bacteria such as *Vibrio cholerae* (Kierek and Watnick, 2003). Calcium ions electrostatically cross-link sodium alginate polymers thereby creating a hydrogel (Sarkisova et al., 2005) and induce aggregation of acidic proteins that comprise the outer layer of the sheath of *Thiothrix*, a different filamentous bacterium (Kawasaki et al., 2018). However, elemental analysis of SP-6 filament sheaths detects only low levels of calcium ions (Kunoh et al., 2017b), which suggests that for SP-6, Ca^2+^ is important in the process of sheath development rather than in the maintenance of the sheath framework.

Although little is known about calcium signaling in bacteria due to their small size and the lack of sensitive tools, Ca^2+^ has been shown to be required for proliferation, chemotaxis, mechanosensing, and spore germination in some bacteria (Tisa and Adler, 1992; Holland et al., 1999; de Vries, 2004), implying that Ca^2+^ could play a role in intracellular signaling in bacteria. Searching the genome sequence database of the SP-6 strain, we find a Na^+^/Ca^2+^ exchanger, which could mean that intracellular Ca^2+^ functions as a signal messenger for sheath development (Brini and Carafoli, 2011). It will be interesting to examine whether Ca^2+^ influx affects the secretion of nanofibrils through gene expression. Furthermore, we concern metagenomic analyses for specifying key metabolite(s) influencing filamentous growth of *Leptothrix* as a future work to advance our present achievement.

### Combinatory Limitation of Carbon Source and Ca^2+^ Breaks Filaments Into Individual Cells

**FIGURE 4:**
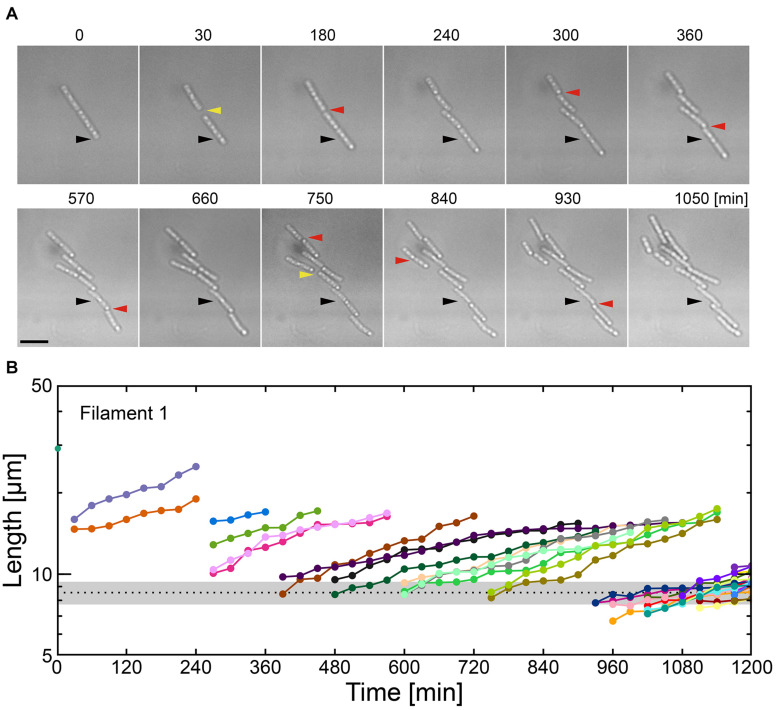
Combinatory limitations of a carbon source and Ca^2+^ causes catastrophic failure of filaments. **(A)** Time-lapse sequences showing frequent filament fracture in MSVP-C-Ca. Black arrowheads indicate the same spatial position in each frame, red arrowheads indicate the locations where the filament breaks, while yellow arrowheads indicate the appearance of intercellular gaps. Scale bar = 5 μm. **(B)** Filament length as a function of time after fracture events corresponding to the cells in **(A)**. The black dotted line indicates the average length of a cell immediately after division in MSVP. (also see Supplementary Figure 11).

Various nutrient limitations have demonstrated dramatic changes to the sheath structure. Severe C-source and Ca^2+^ restrictions, respectively, have produced some of the most dramatic phenotypic changes to filamentous growth. We track the development of SP-6 cell filaments cultured in MSVP-C-Ca in the 2D chambers to find that after a few divisions, the resultant daughter cells appear to be free of any connections to their siblings due to the combinatory production of intercellular gaps (yellow arrowheads) and breaks (red arrowheads) (Figure 4A and Supplementary Movie 12). By tracking filament length, we find a steady decrease in the average filament length over time to the point where multicellular filaments disappear completely leaving only individual dividing cells, as shown in Figure 4B. This contrasts with fragmentation that occurs in MSVP-Ca and MSVP + EGTA, where the smallest filaments still contain at least two cells (Figures 3B, 4B and Supplementary Figure 11). This result demonstrates that combined limitation can completely suppress filamentation by targeting different required components of sheath construction used by *Leptothrix*. Importantly, although the rate of filament elongation slows, it is not arrested in this medium, similar to the case of MSVP-Ca (Figure 3C). This suggests that the combined limitation is effective in completely suppressing filamentous growth, while not interfering with cell growth.

### Abrogation of Precursors to Floating Microbial Mats by Combinatory Limitation of a Carbon Source and Ca^2+^

**FIGURE 5:**
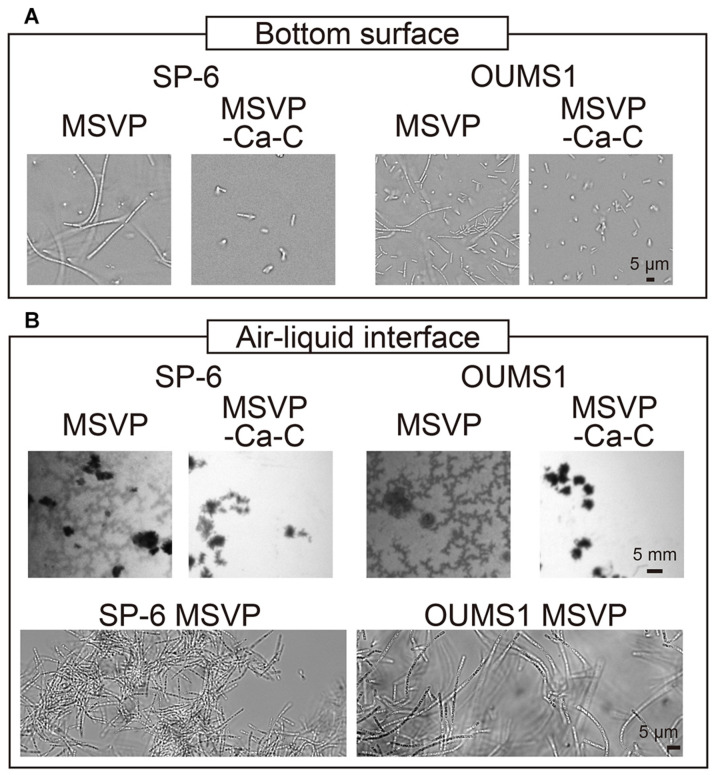
Growth of SP-6 and OUMS1 cells in stationary liquid media. Images of SP-6 and OUMS1 cells after 24 h incubation and 12 h incubation, respectively, at the bottom of the dish **(A)** and the air-liquid interface **(B)**. The second set of micrographs in **(B)** are bright-field images of aggregates that were scooped out and imaged. Images of the cells taken in MSVP and MSVP-C-Ca are shown side by side for comparison.

Our 2D microfluidic chambers enables us to screen the effect of various nutrient conditions on the innate behavior of *Leptothrix* cells, while simultaneously enabling the capture of single-cell and single-filament level images. However, to examine the hierarchical effects of nutrient limitation, we grow filaments in a 3D environment. We culture SP-6 cells in polymer coverslip bottom dishes using either MSVP or MSVP-C-Ca and record time-lapse images (Figure 5A, left, Supplementary Movie 13). After 24 h incubation in MSVP, we observe that a number of cells at the bottom surface form filaments. In contrast, over the same time span, cells cultured in MSVP-C-Ca result in numerous surface-attached cells and an obvious increase in the number of planktonic cells, however, there are no filaments. These results suggest that combinatory limitation does not interfere with the cell division but strongly affects filament formation, a necessary step in producing a network of woven cell filaments.

*Leptothrix* species are known to prefer aerobic conditions. *L. ochracea*, which was not used in the present work, is known to produce floating mats that consist of woven cell filaments in stagnant water (Kunoh et al., 2016b). Similarly, we observe the formation of thin films consisting of entangled filaments at the air-liquid interface when SP-6 cells are cultured under static conditions in MSVP, whereas culture in MSVP-C-Ca ablates the floating films (Figure 5B, left, Supplementary Movie 14).

To test whether the effects of calcium and carbon depletion on SP-6 is a general phenomenon, we use the OUMS1 strain of *L. cholodnii*. We culture OUMS1 cells in either MSVP or MSVP-C-Ca, tracking the aggregation of cell filaments in polymer coverslip bottom dishes. Similar to the SP-6 cells, after a 12 h incubation we find that OUMS1 cells aggregate to form filaments at the bottom surface in MSVP, while in MSVP-C-Ca, OUMS1 remain as individual cells similar to SP-6 cultured under the same conditions (Figure 5A, right). Moreover, OUMS1 cells cultured in static conditions in MSVP produce a floating film, whereas this is not observed in dishes containing MSVP-C-Ca (Figure 5B, right). Based on these data, we conclude that the effect on filamentation in *Leptothrix* due to depletion of carbon and calcium is a conserved property across at least two related strains and may be a more general phenomenon.

## Conclusion and Outlook

The microbial mats produced by *Leptothrix* are complex 3D structures composed of entangled cell filaments; this random woven structure makes real-time analysis of single-cells in individual filaments difficult. In this work, we use 2D microfluidic chambers to enable acquisition of movies of individual cell filament growth at high spatial and temporal resolution. Microfluidics enables easy addition of fluorescent labeling reagents, chemical treatment, and medium change without disturbing the growing filaments. With this platform, we show four modes of filamentous growth induced by nutrient limitation: normal (Na^+^, K^+^, and Fe^2+^), patchy (carbon, nitrogen, phosphorus, and vitamins), non-viable (Mg^2+^), and fragmented (Ca^2+^).

Importantly, colimitation of Ca^2+^ and C sources leads to the occurrence of planktonic cells, however, without dramatic effects on cell division. This suggests a potential method to fragment cell filaments by utilizing combinatory nutrient limitations to induce particular phenotypic modes. Our findings may provide industry with additional tools to manage filamentous growth by regulating the causes of filament growth rather than only treating their effects, although larger scale tests will be necessary. It is worth utilizing chelators and flocculants for combinatory removal of nutrients such as divalent cations (Mg^2+^ and Ca^2+^) and phosphorus.

## Data Availability Statement

The raw data supporting the conclusions of this article will be made available by the authors, without undue reservation.

## Author Contributions

TK, TY, NN, and AU designed the study. TK, TY, SS, and EO acquired the data. TK, TY, and AU analyzed and interpreted the data. TK and AU wrote the manuscript. All authors agreed to submit the manuscript.

## Conflict of Interest

The authors declare that the research was conducted in the absence of any commercial or financial relationships that could be construed as a potential conflict of interest.

## Footnotes

1 https://www.r-project.org/

## Supplementary Material

Supplementary Figure 1 Typical cell filament elongation in MSVP.

Supplementary Figure 2 Cell filament elongation in MSVP-K.

Supplementary Figure 3 Cell filament elongation in MSVP-Fe.

Supplementary Figure 4 Time-lapse image sequence showing filamentous growth in MSVP-N, MSVP-P, and MSVP-V within the 2D chamber.

Supplementary Figure 5 The percentage of the filament filled with cells in MSVP-C, MSVP-N, MSVP-P, and MSVP-V.

Supplementary Figure 6 Fluorescent labeling of the sheath n MSVP-N, MSVP-P, and MSVP-V.

Supplementary Figure 7 ASEM snapshots of SP-6 cell filaments cultured in MSVP and MSVP-lowC.

Supplementary Figure 8 Autolytic cell death in MSVP-Mg.

Supplementary Figure 9 Characteristic elongation of cell filaments in MSVP-Ca.

Supplementary Figure 10 Cell filament breakage in MSVP-Ca(NH_4_Cl) medium.

Supplementary Figure 11 Cell filament elongation before and after EGTA treatment.

Supplementary Figure 12 Cell filament breakage caused by the EGTA treatment in 2xPYG medium.

Supplementary Figure 13 Combinatory limitations of a carbon source and Ca^2+^ stimulate planktonic cell generation.

Supplementary Table 1 Composition of MSVP medium.

Supplementary Table 2 Composition of vitamin stock solution for MSVP medium.

Supplementary Movie 1 Time-lapse video of SP-6 cells cultured in MSVP (mp4).

Supplementary Movie 2 Time-lapse video of SP-6 cells cultured in MSVP-Na (mp4).

Supplementary Movie 3 Time-lapse video of SP-6 cells cultured in MSVP-K (mp4).

Supplementary Movie 4 Time-lapse video of SP-6 cells cultured in MSVP-Fe (mp4).

Supplementary Movie 5 Time-lapse video of SP-6 cells cultured in MSVP-C (mp4).

Supplementary Movie 6 Time-lapse video of SP-6 cells cultured in MSVP-N (mp4).

Supplementary Movie 7 Time-lapse video of SP-6 cells cultured in MSVP-P (mp4).

Supplementary Movie 8 Time-lapse video of SP-6 cells cultured in MSVP-V (mp4).

Supplementary Movie 9 Time-lapse video of SP-6 cells cultured in MSVP-Mg (mp4).

Supplementary Movie 10 Time-lapse video of SP-6 cells cultured in MSVP-Ca (mp4).

Supplementary Movie 11 Time-lapse video of SP-6 cells before **(A)** and after **(B)** the addition of EGTA to the original MSVP during culturing (mp4).

Supplementary Movie 12 Time-lapse video of SP-6 cells cultured in MSVP-C-Ca (mp4).

Supplementary Movie 13 Time-lapse video of SP-6 cells at the bottom surface of a polymer coverslip bottom dish in MSVP (left) and MSVP-C-Ca (right) (mp4).

Supplementary Movie 14 Time-lapse video of SP-6 cells in MSVP (left) and MSVP-C-Ca (right) at the air-liquid interface of a glass bottom dish (mp4).
