# Supplementary material for: Leptothrix cholodnii Response to Nutrient Limitation
Source: Front Microbiol. 2021 Jun 24;12:691563. doi: 10.3389/fmicb.2021.691563 (PMC8264430; doi:10.3389/fmicb.2021.691563)
Supplement: Supplementary Figure 1 — Typical cell filament elongation in MSVP. [file Data_Sheet_1.docx]

Supplementary Material

# Supplementary Data

Supplementary movies are available at FigShare and received

https://figshare.com/articles/media/MovieS1_mp4/14566707/1

Movie S1: Time-lapse video of SP-6 cells cultured in MSVP (mp4).

Movie S2: Time-lapse video of SP-6 cells cultured in MSVP-Na (mp4).

Movie S3: Time-lapse video of SP-6 cells cultured in MSVP-K (mp4).

Movie S4: Time-lapse video of SP-6 cells cultured in MSVP-Fe (mp4).

Movie S5: Time-lapse video of SP-6 cells cultured in MSVP-C (mp4).

Movie S6: Time-lapse video of SP-6 cells cultured in MSVP-N (mp4).

Movie S7: Time-lapse video of SP-6 cells cultured in MSVP-P (mp4).

Movie S8: Time-lapse video of SP-6 cells cultured in MSVP-V (mp4).

Movie S9: Time-lapse video of SP-6 cells cultured in MSVP-Mg (mp4).

Movie S10: Time-lapse video of SP-6 cells cultured in MSVP-Ca (mp4).

Movie S11: Time-lapse video of SP-6 cells before (A) and after (B) the addition of EGTA to the original MSVP during culturing (mp4).

Movie S12: Time-lapse video of SP-6 cells cultured in MSVP-C-Ca (mp4).

Movie S13: Time-lapse video of SP-6 cells at the bottom surface of a polymer coverslip bottom dish in MSVP (left) and MSVP-C-Ca (right) (mp4).

Movie S14: Time-lapse video of SP-6 cells in MSVP (left) and MSVP-C-Ca (right) at the air-liquid interface of a glass bottom dish (mp4).

# Supplementary Figures and Tables

##
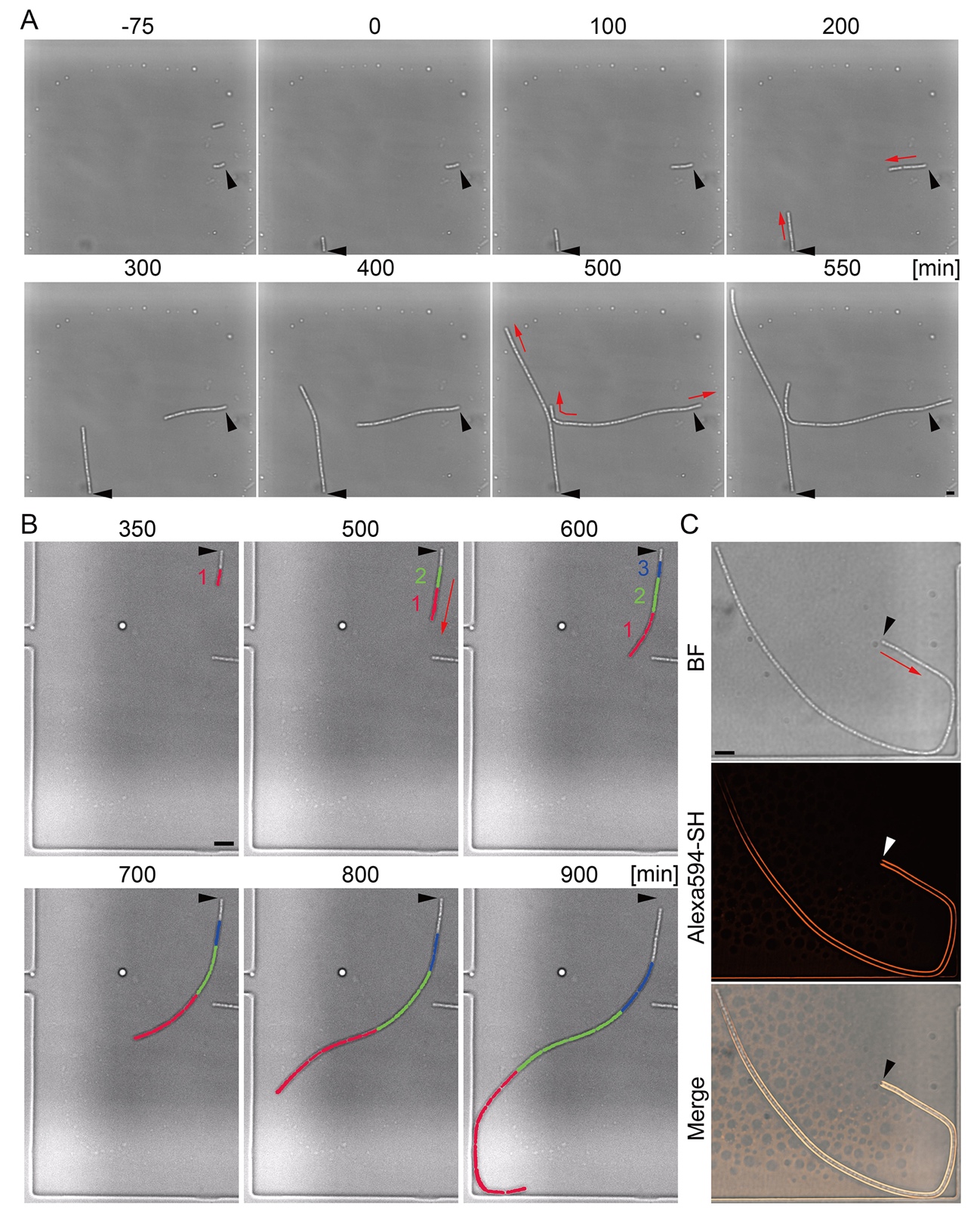
Supplementary Figures

**Supplementary Figure 1.** Typical cell filament elongation in MSVP. (A) Time-lapse image sequence of filamentous growth after surface attachment (*t* = 0 min). (B) Continuous division of cells in the cell chain. After the initial division at *t* = 350 min, the leading daughter cell and its descendants are all colored red, while the second division of the surface-attached daughter cell (and its decedents) are colored green. The third division of the attached daughter cell (and its decedents) is colored blue. (C) Fluorescent labeling of the sheath. Black and white arrowheads indicate the same spatial positions in each set of frames, while red arrows indicate the direction of elongation. Scale bars = 5 *μ*m.


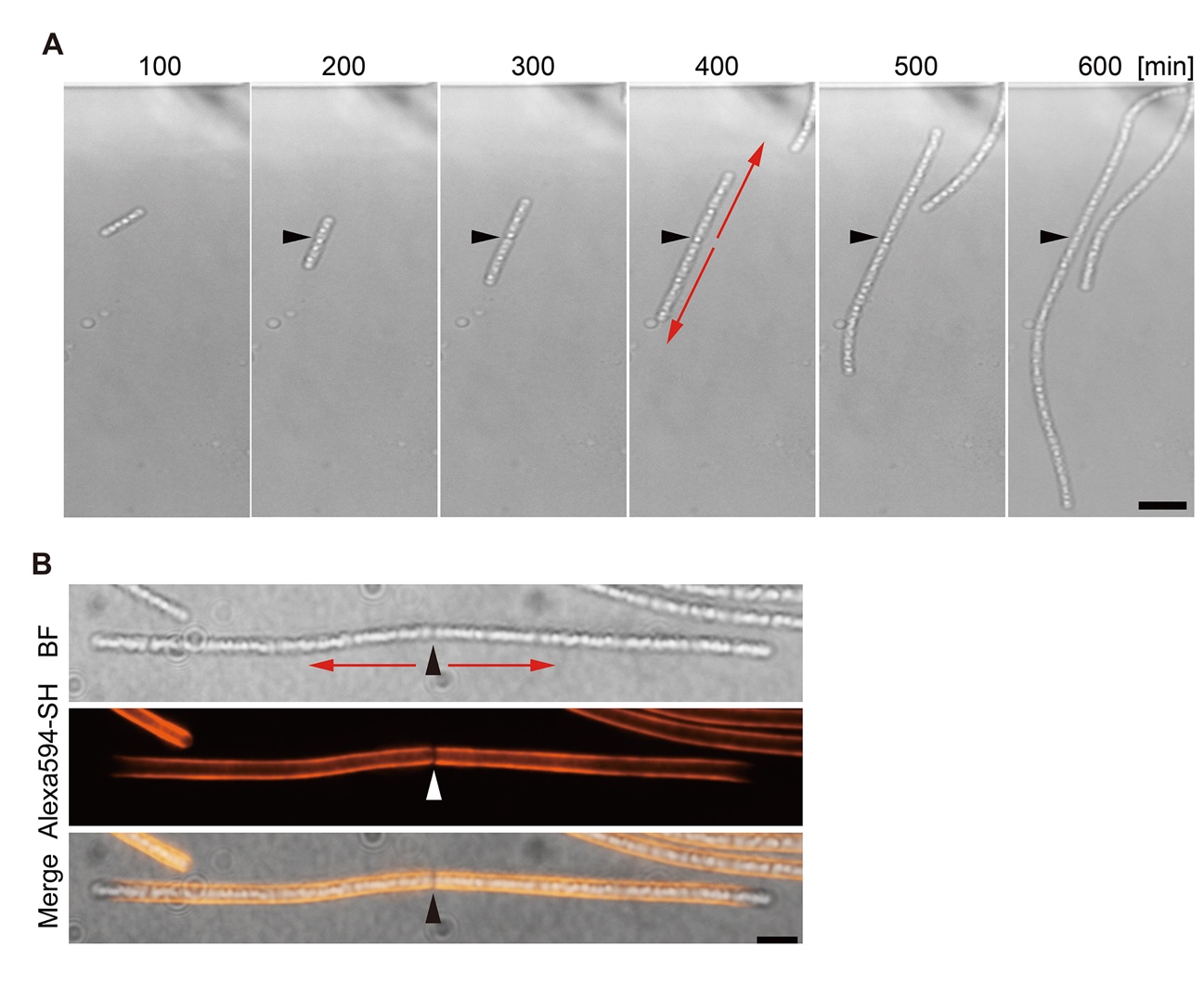


**Supplementary Figure 2.** Cell filament elongation in MSVP-K. (A) Time-lapse image sequence showing filamentous growth within the 2D chamber. (B) Fluorescent labeling of the sheath. Black and white arrowheads indicate the same spatial position in each frame, while red arrows indicate the direction of elongation. Scale bars = 5 *μ*m.

**
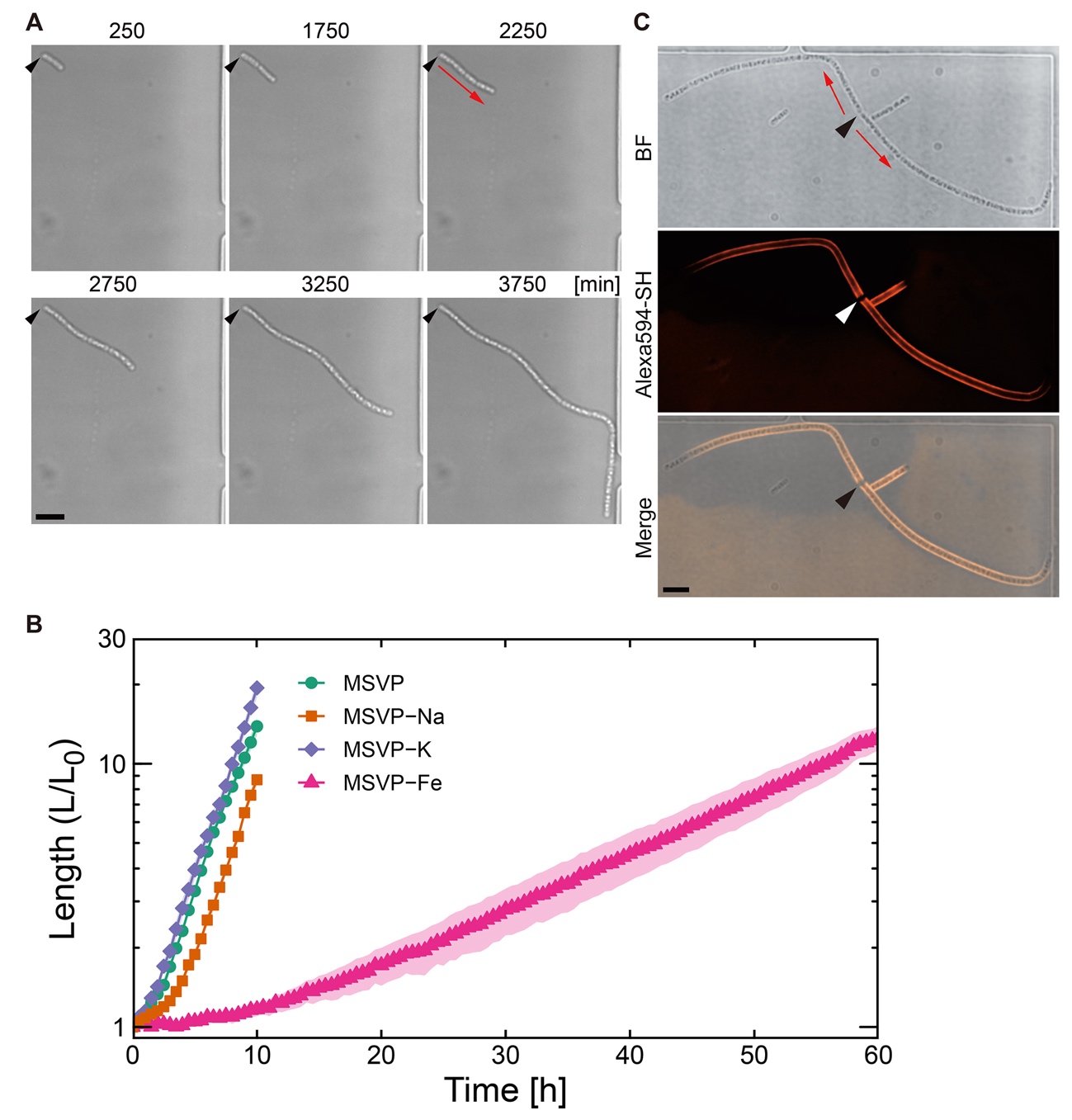
**

**Supplementary Figure 3.** Cell filament elongation in MSVP-Fe. (A) Time-lapse image sequence showing filamentous growth within the 2D chamber. (B) Average length (dotted line) and SD (filled region) of three filaments in MSVP-Fe (magenta), compared to filaments growing in MSVP (green), MSVP-Na (orange), MSVP-K (purple). (C) Fluorescent labeling of the sheath. Black and white arrowheads indicate the same spatial position in each set of frames, while red arrows indicate the direction of elongation. Scale bars = 5 *μ*m.


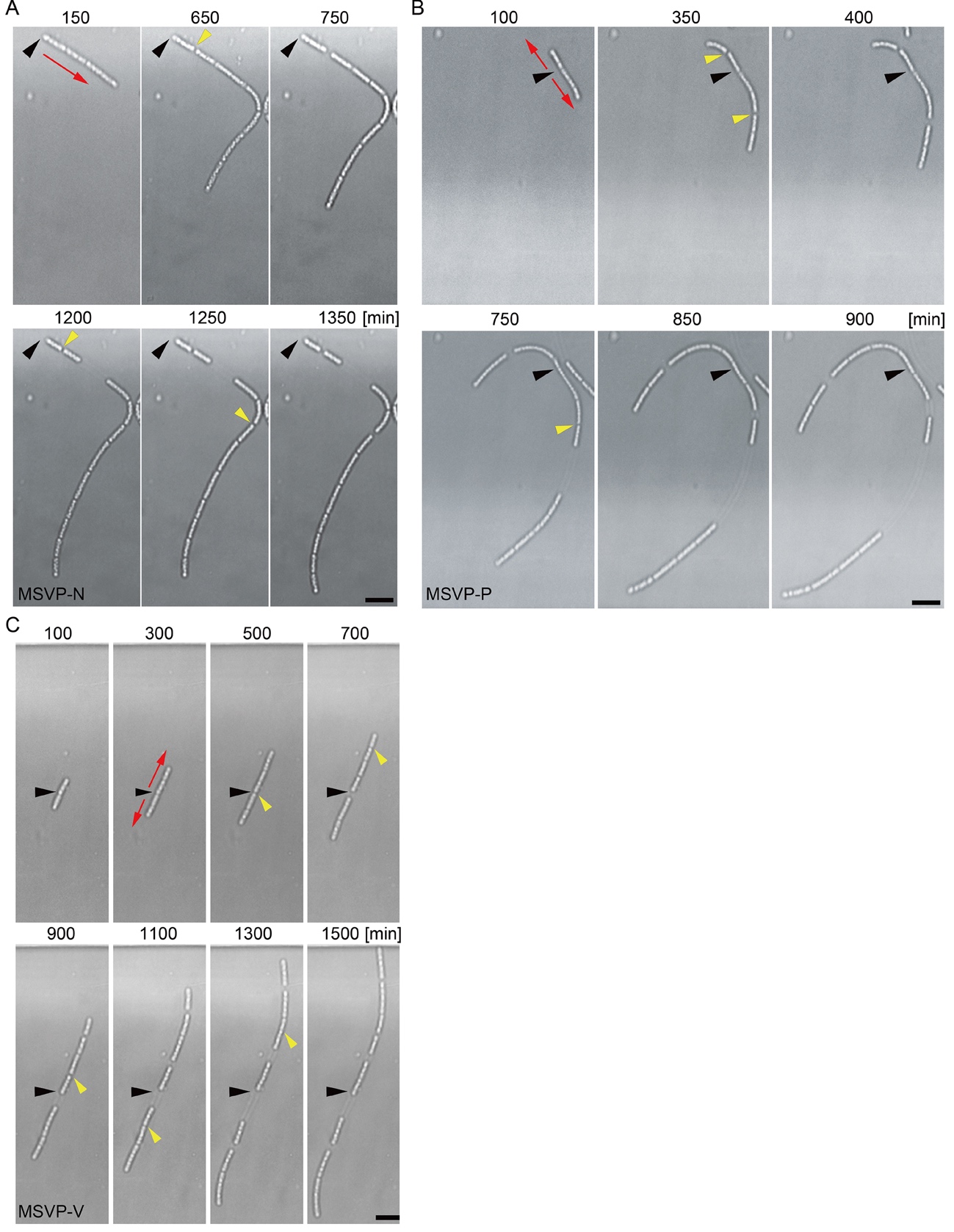


**Supplementary Figure 4.** Time-lapse image sequence showing filamentous growth in MSVP-N (A), MSVP-P (B), and MSVP-V (C) within the 2D chamber. Black arrowheads indicate the same spatial position in each set of frames, while red arrows and yellow arrowheads indicate the direction of elongation and the positions of wide intercellular gaps, respectively. Scale bars = 5 *μ*m.


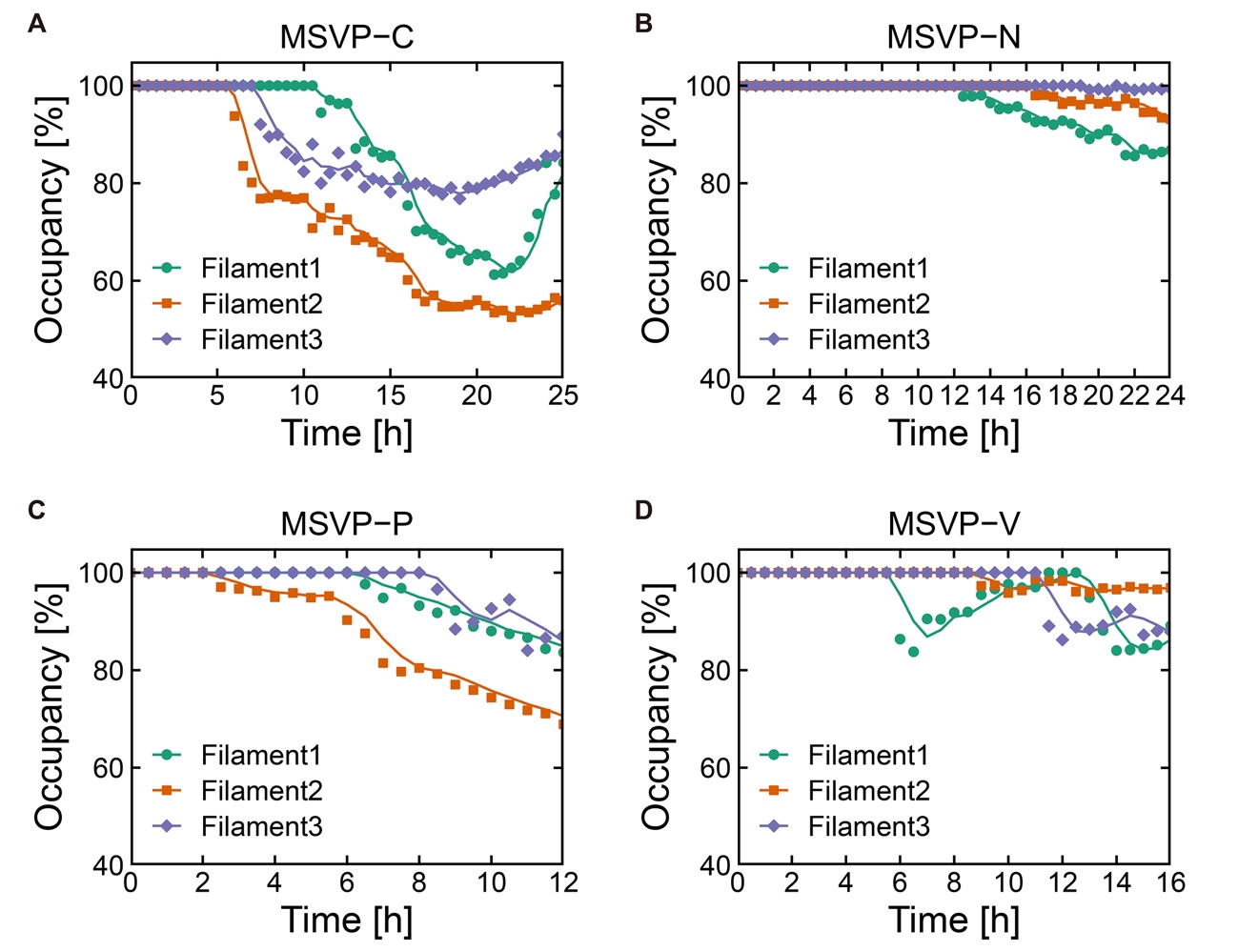


**Supplementary Figure 5.** The percentage of the filament filled with cells in MSVP-C (A), MSVP-N (B), MSVP-P (C), and MSVP-V (D) as a function of time measured for three separate filaments, labeled Filament 1-3 (see also Figure 2D).


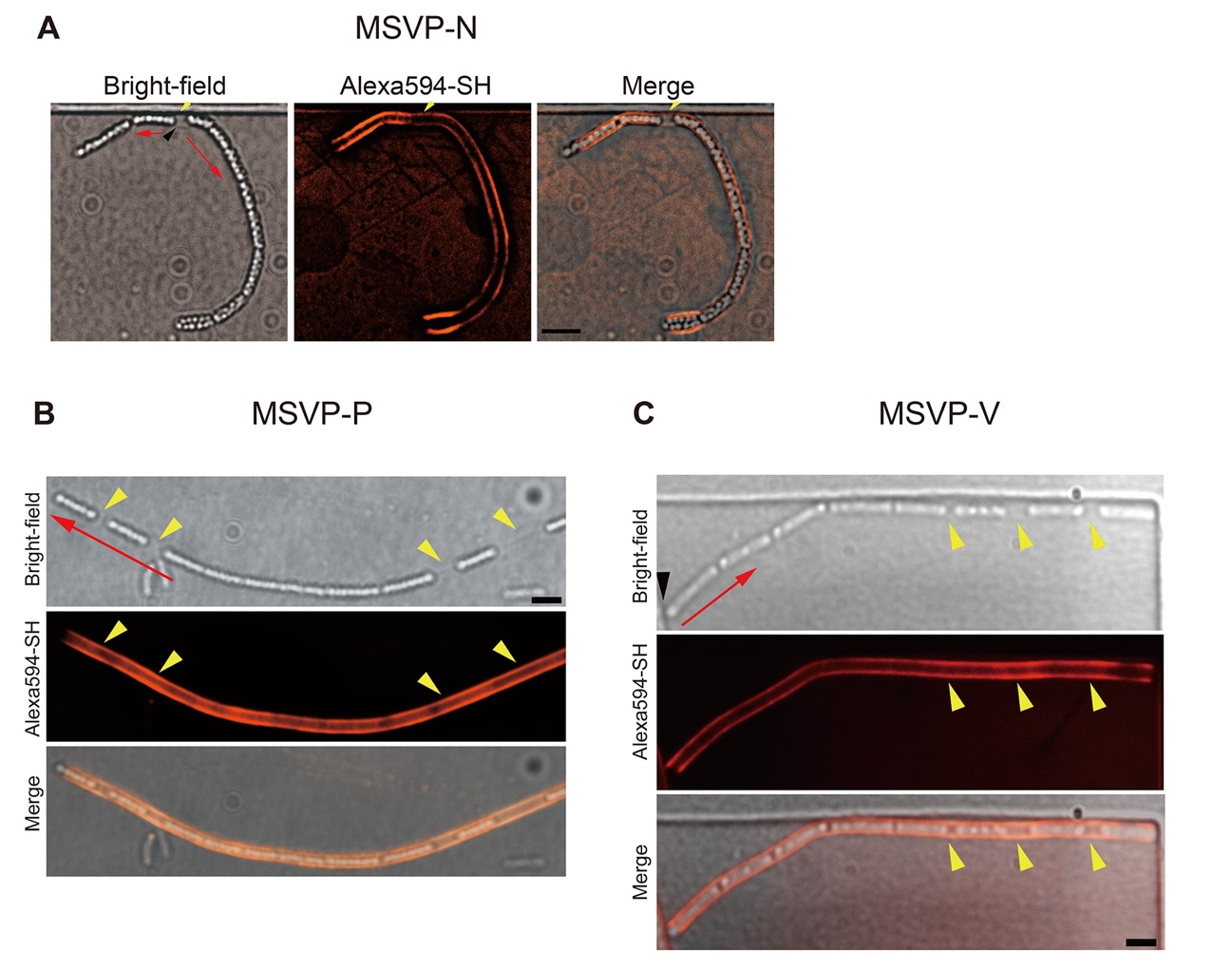


**Supplementary**
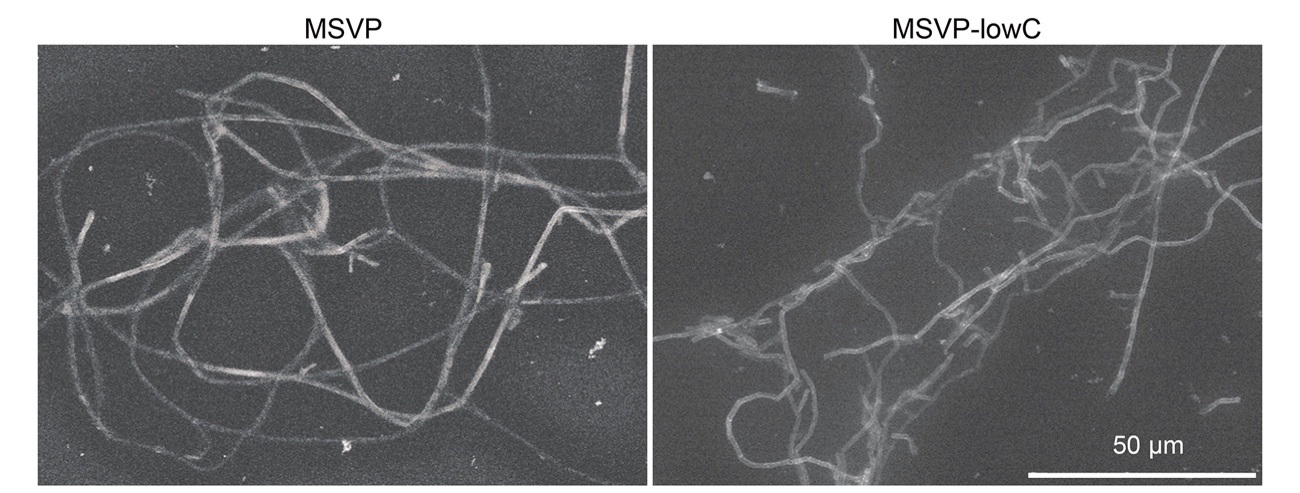
**Figure 6.** Fluorescent labeling of the sheath n MSVP-N (A), MSVP-P (B), and MSVP-V (C). Black and white arrowheads indicate the same spatial position in each set of frames, while red arrows and yellow arrowheads indicate the direction of elongation and the positions of wide intercellular gaps, respectively. Scale bars = 5 *μ*m

**Supplementary Figure 7.** ASEM snapshots of SP-6 cell filaments cultured in MSVP (left) and MSVP-lowC (right).


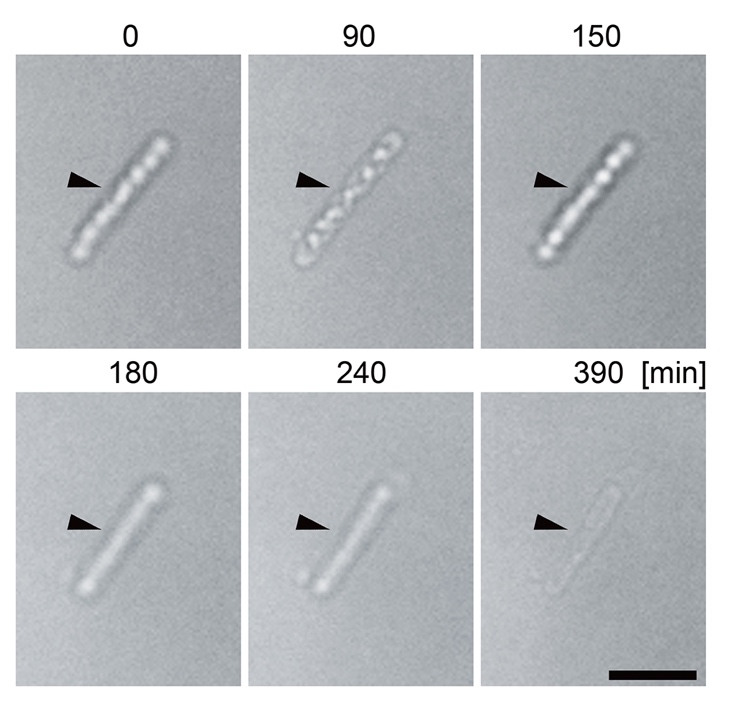


**Supplementary Figure 8.** Autolytic cell death in MSVP-Mg. Time-lapse image sequence showing cell death within the 2D chamber. Black arrowheads indicate the same spatial position in each frame. Scale bar = 5 *μ*m.


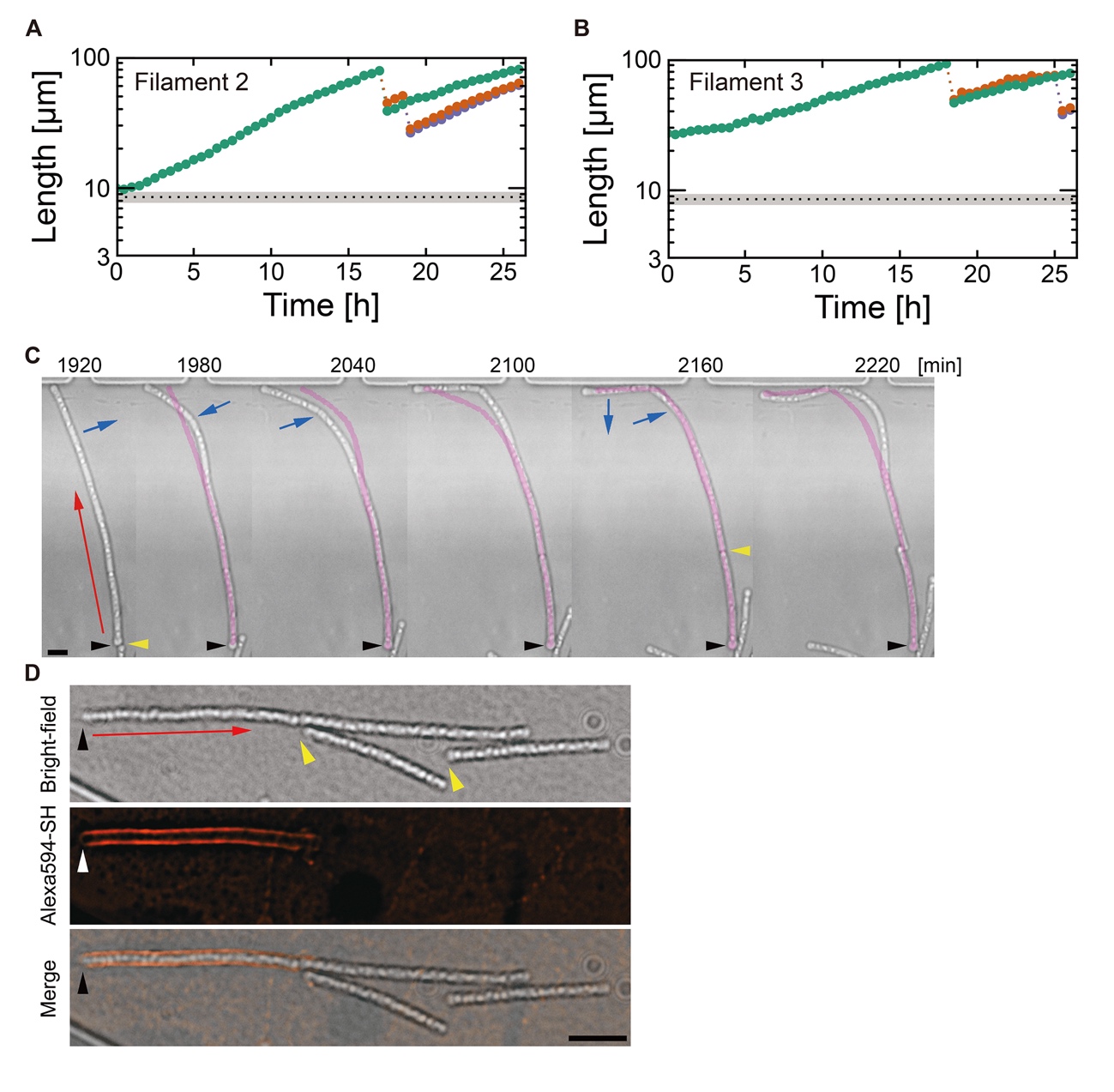


**Supplementary Figure 9.** Characteristic elongation of cell filaments in MSVP-Ca. (A,B) Filament elongation and fragmentation as a function of time for two separate filaments. Elongating mother filaments fragment into daughter filaments designated by the vertical dotted line. The black dotted lines indicate the average length of a cell immediately after division in MSVP. (C) Time-lapse image sequences showing filament bending after impact with the chamber wall (*t* = 1920 min). The magenta line is the trace of the filament from the preceding frame. It is superimposed on the bright-field image to enable comparison. (D) Fluorescent labeling of the sheath. Black and white arrowheads indicate the same spatial position in each set of frames, red and blue arrows indicate the direction of elongation and bending, respectively, and yellow arrowheads indicate filament fracture points in (C, D). Scale bars = 5 *μ*m.

~~
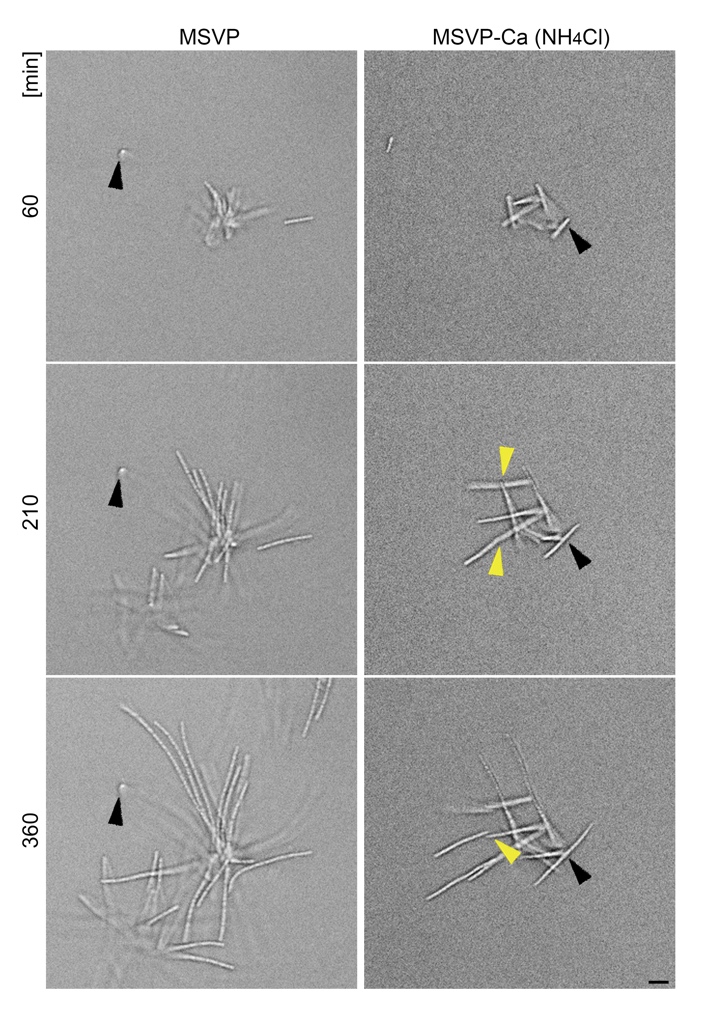
~~**Supplementary Figure 10.** Cell filament breakage in MSVP-Ca(NH_4_Cl) medium. Time-lapse image sequence showing filamentous growth in control MSVP (left) and MSVP-Ca (NH_4_Cl) (right) medium. Initial surface attachment of the cells is defined as *t* = 0. Black arrowheads indicate the same spatial position in each frame and yellow arrowheads indicate points where the filament breaks. Scale bars = 5 *μ*m.


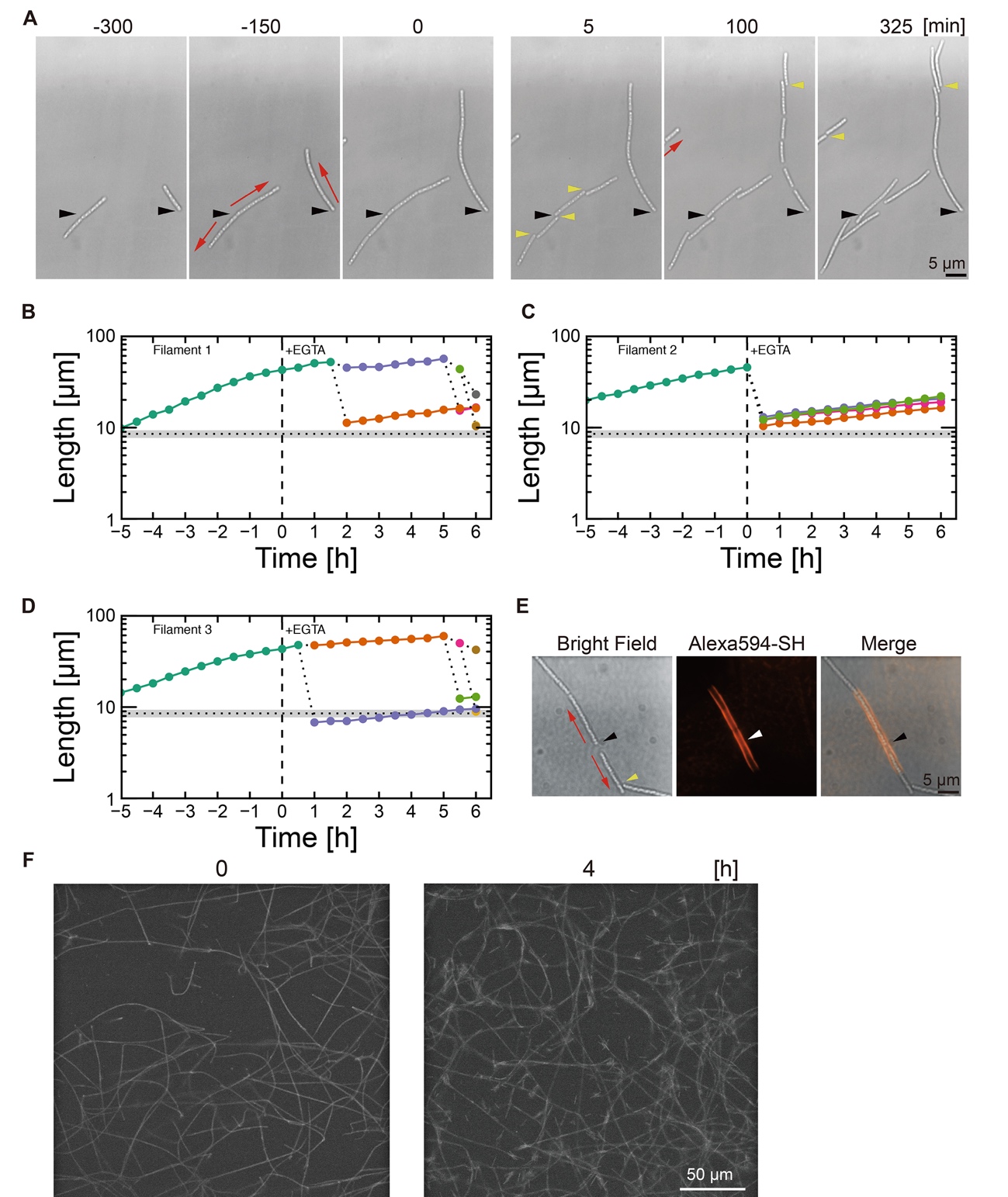


**Supplementary Figure 11.** Cell filament elongation before and after EGTA treatment. (A) Time-lapse image sequences showing filamentous growth in MSVP before and after the treatment. The time of the addition of EGTA is defined as *t* = 0 min. (B-D) Filament length of three independent filaments measured as a function of time. The black dotted lines indicate the average length of a cell immediately after division in MSVP. (E) Fluorescent images showing sheath distribution 4 h after the addition of EGTA. Black and white arrowheads in (A,E) indicate the same spatial position in each frame. Red arrows and yellow arrowheads indicate the direction of elongation and points where the filament breaks. (F) ASEM snapshots of cell filaments before (0 h) and after (4 h) the addition of EGTA.


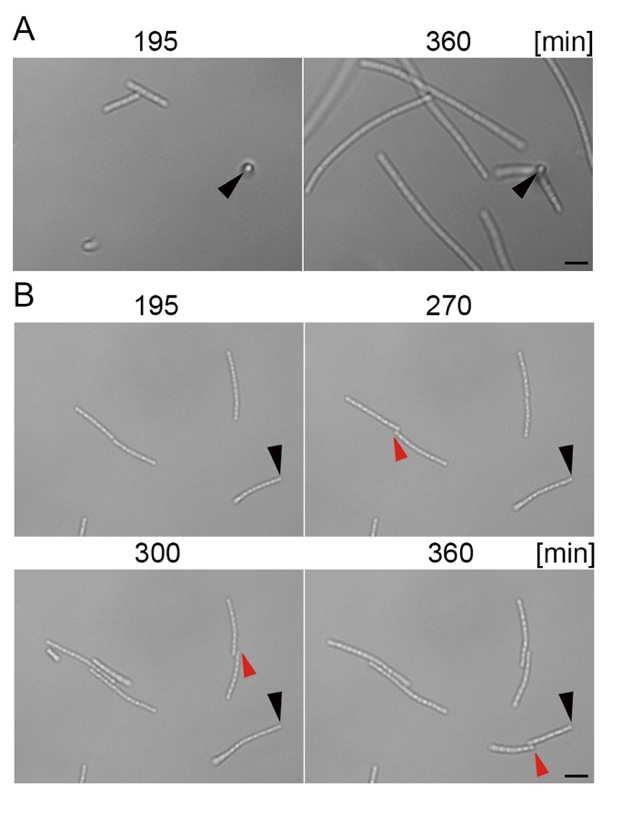
**Supplementary Figure 12.** Cell filament breakage caused by the EGTA treatment in 2xPYG medium. Time-lapse image sequence showing filamentous growth in the control 2xPYG (A) and 2xPYG supplemented with 0.5 mM EGTA (B). Initial surface attachment of the cells is defined as *t* = 0. Black arrowheads indicate the same spatial position in each frame and red arrowheads indicate points where the filament breaks. Scale bars = 5 *μ*m.


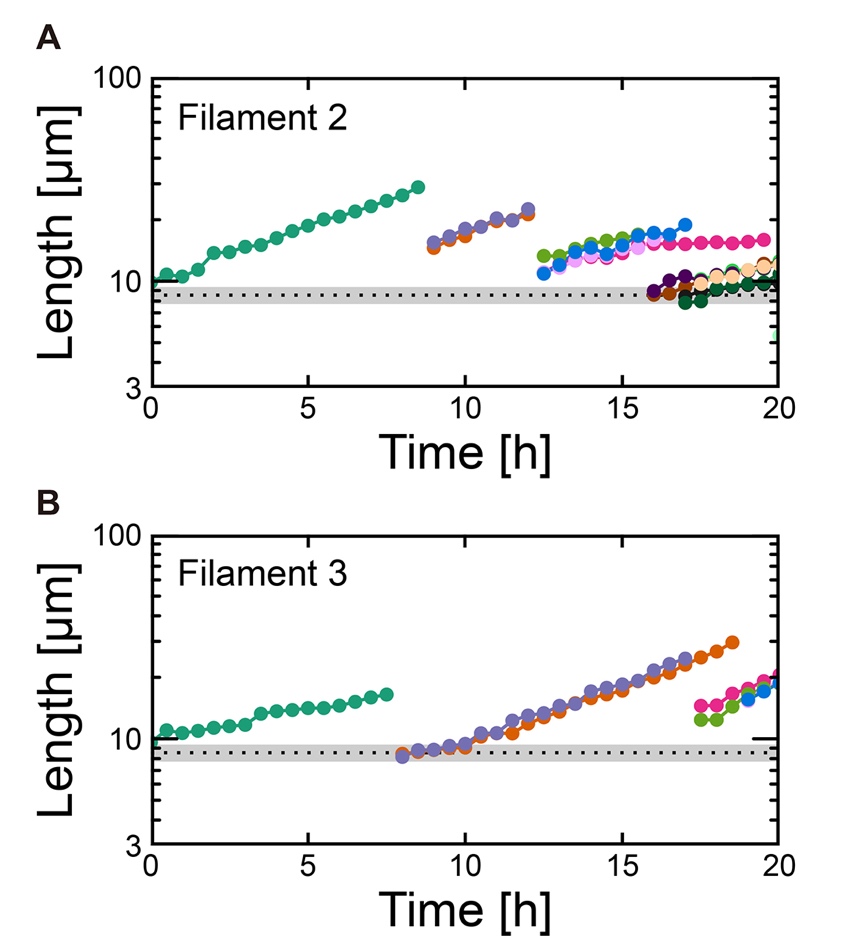
**Supplementary Figure 13.** Combinatory limitations of a carbon source and Ca^2+^ stimulate planktonic cell generation. The lengths of daughter filaments are plotted as a function of time after fracture events. The black dotted lines indicate the average length of a cell immediately after division in MSVP. (also see Figure 4B).

## Supplementary Tables

**Supplementary Table 1.** Composition of MSVP medium.

**Supplementary Table 2.** Composition of vitamin stock solution for MSVP medium.
